# Effectiveness of a Nursing Intervention to Improve Knowledge, Attitudes, and Practices in Malaria Prevention in an Emberá Katío Community in the Department of Córdoba

**DOI:** 10.17533/udea.iee.v42n3e11

**Published:** 2024-10-25

**Authors:** Jorge Luis Herrera Herrera, María de los Ángeles Rodríguez-Gázquez, Juan Guillermo Rojas

**Affiliations:** 1 RN, Ph.D. candidate. Associated Professor at Program of Nursing, Universidad de Córdoba, Colombia. Email: jluisherrera@correo.unicordoba.edu.co. Corresponding author. https://orcid.org/0000-0001-9738-6891 Universidad de Córdoba Program of Nursing Universidad de Córdoba Colombia jluisherrera@correo.unicordoba.edu.co; 2 RN, Ph.D. Full Professor at Faculty of Nursing, Universidad de Antioquia, Colombia. Email: maria.rodriguezg@udea.edu.co. https://orcid.org/0000-0002-4329-4286 Universidad de Antioquia Faculty of Nursing Universidad de Antioquia Colombia maria.rodriguezg@udea.edu.co; 3 RN, Ph.D. Full Professor at Faculty of Nursing, Universidad de Antioquia, Colombia. Email: guillermo.rojas@udea.edu.co https://orcid.org/0000-0002-0255-4344 Universidad de Antioquia Faculty of Nursing Universidad de Antioquia Colombia guillermo.rojas@udea.edu.co

**Keywords:** malaria, health knowledge, attitudes, practice, indigenous peoples, transcultural nursing, malaria, conocimientos, actitudes y prácticas en salud, pueblos indígenas, enfermería transcultural, malária, conhecimentos, atitudes e prática em saúde, povos indígenas, enfermagem transcultural.

## Abstract

**Objective.:**

To evaluate the effectiveness of a nursing intervention, against routine care, to improve knowledge, attitudes, and practices (KAP) in malaria prevention in an Emberá Katío community from the department of Córdoba, Colombia.

**Methods.:**

This was an intervention study with quasi-experimental design with control group, conducted in three phases: (I) design of the educational intervention, (II) content validation of the educational intervention through expert judgment, and (III) execution of a quasi-experimental study with two groups: experimental (*n* = 60) and control (*n* = 58). The intervention consisted in four modules taught in person, using educational strategies, like classes, guided discussions, workshops, and a booklet designed for the study. The control group received the routine care provided by the Secretariat of Health. The study used the instrument by the Pan-American Health Organization “Survey on knowledge, attitudes, and practices in addressing malaria in indigenous communities” to measure pre- and post-intervention scores.

**Results.:**

The four modules of the educational intervention obtained Content Validity Indices between 0.83 and 0.90 that are considered adequate. The General Linear Models of repeated measures showed positive effect of the educational intervention on the KAP scores (*p* < 0.001), with an effect size of 91% in knowledge, 49% in attitudes, 85% in practices, and 93% in the total score.

**Conclusion.:**

The educational intervention proved effective to improve KAPs in malaria prevention in the Emberá Katío community from the department of Córdoba.

## Resumen

**Objetivo.:**

Evaluar la efectividad de una intervención de enfermería, frente a la atención rutinaria, para mejorar los conocimientos, actitudes y prácticas (CAP) en la prevención de la malaria en una comunidad emberá katío del departamento de Córdoba, Colombia.

**Métodos.:**

Estudio de intervención con diseño cuasiexperimental con grupo control, realizado en tres fases: (I) diseño de la intervención educativa, (II) validación del contenido de la intervención educativa mediante juicio de expertos, y (III) ejecución de un estudio cuasiexperimental con dos grupos: experimental (*n* = 60) y control (*n* = 58). La intervención consistió en cuatro módulos impartidos presencialmente, utilizando estrategias educativas como clases, discusiones guiadas, talleres y una cartilla diseñada para el estudio. El grupo control recibió la atención rutinaria proporcionada por la Secretaría de Salud. Se utilizó el instrumento de la Organización Panamericana de la Salud “Encuesta sobre conocimientos, actitudes y prácticas en el abordaje de la malaria en comunidades indígenas” para medir los puntajes pre- y post-intervención.

**Resultados.:**

Los cuatro módulos de la intervención educativa obtuvieron Índices de Validez de Contenido entre 0.83 y 0.90 que se consideran adecuados. Los modelos lineales generales de medidas repetidas mostraron un efecto positivo de la intervención educativa en los puntajes de CAP (*p* < 0.001), con un tamaño del efecto del 91 % en conocimientos, 49 % en actitudes, 85 % en prácticas y 93 % en el puntaje total.

**Conclusión.:**

La intervención educativa demostró ser efectiva para mejorar los CAP en la prevención de la malaria en la comunidad emberá katío del departamento de Córdoba.

## Resumo

**Objetivo.:**

Avaliar a eficácia de uma intervenção de enfermagem, comparada aos cuidados de rotina, para melhorar conhecimentos, atitudes e práticas (CAP) na prevenção da malária em uma comunidade Embera Katío, no departamento de Córdoba, Colômbia.

**Métodos.:**

Estudo de intervenção com desenho quase experimental com grupo controle, realizado em três fases: (I) desenho da intervenção educativa, (II) validação do conteúdo da intervenção educativa por meio de julgamento de especialistas, e (III) execução de um estudo quase experimental com dois grupos: experimental (*n* = 60) e controle (*n* = 58). A intervenção consistiu em quatro módulos ministrados presencialmente, utilizando estratégias educativas como aulas, discussões orientadas, oficinas e uma cartilha elaborada para o estudo. O grupo controle recebeu cuidados de rotina fornecidos pelo Ministério da Saúde. O instrumento da Organização Pan-Americana da Saúde “Pesquisa sobre conhecimentos, atitudes e práticas no enfrentamento da malária em comunidades indígenas” foi utilizado para medir as pontuações pré e pós-intervenção.

**Resultados.:**

Os quatro módulos da intervenção educativa obtiveram Índices de Validade de Conteúdo entre 0.83 e 0.90, considerados adequados. Modelos lineares gerais de medidas repetidas mostraram efeito positivo da intervenção educativa nos escores do PAC (*p* < 0.001), com tamanho de efeito de 91% para conhecimento, 49% para atitudes, 85% para práticas e 93% para escore total.

**Conclusão.:**

A intervenção educativa revelou-se eficaz na melhoria do PAC na prevenção da malária na comunidade Embera Katío do departamento de Córdoba.

## Introduction

Malaria or paludism is a potentially deadly disease caused by protozoans of the genus *Plasmodium,* transmitted to humans through the bite from female mosquitos from the genus *Anopheles*.^1^ This disease is the most-common parasitic infection in the world with 249-million cases notified and 608 000 deaths due to this cause in 2022.^2^ Colombia is no stranger to this problem, with 102,455 cases of paludism registered in 2023, of which one in every four affected belonged to indigenous communities.^3^ For the same year, the department of Córdoba notified 15150 cases of malaria, with Tierralta being one of the municipalities most affected.^4^ In this municipality, located in the Alto Sinú region, the settlement of the Emberá Katío people is located, with 48,117 indigenous people in Colombia, thus, representing 2.7% of the country’s indigenous population.^5^

When analyzing the literature related with strategies used for malaria prevention and the consequential decrease of this disease’s incidence, it is noted that surveys about knowledge, attitudes, and practices (KAP) permit determining in a population the degree of knowledge about malaria, as well as the attitudes and practices that contribute to its transmission; with the results from these surveys being highly important because they can be used to design educational interventions in indigenous communities.^6^ Examples of the foregoing are the KAP surveys conducted in Venezuela,^7^ Panama,^8^ and Colombia,^9^^,^^10^ which showed that culturally adapted educational interventions improved not only knowledge, but achieved changes in attitudes and practices to diminish the risk of suffering this disease.^11^ In this sense, the Pan-American Health Organization^12^ recognizes that an intercultural approach promotes equal treatment of the different cultural groups. Likewise, it considers health a fundamental right, advising that health professionals, among them those from nursing, must lead educational activities with communities to promote integration of traditional KAPs regarding the disease. In this study, the concept of cultural competence was taken from the nursing discipline, which permitted articulating and testing empirically the assumptions or theoretical proposals selected from the model proposed by Rachel Spector.^13^ Similarly, in the literature review, prior to the development of this research, a void was identified in knowledge related to what would be the effectiveness of an educational intervention for malaria prevention focused on KAPs, constructed bearing in mind the cultural issues of care, incorporating in its design theoretical elements from cultural competence. This gave way to conducting this study that sought to evaluate the effectiveness of a nursing intervention against routine care to improve KAPs in malaria prevention in indigenous people of the Emberá Katío ethnic group of the department of Córdoba.

## Methods

The study was carried out in three stages: 

*Stage 1- Design of the educational intervention*

From the review of articles about educational interventions to improve KAPs regarding malaria prevention in indigenous communities, an educational intervention was designed that was initially presented to the leaders of the community object of study, who provided their input and knowledge and which were incorporated to the final intervention. This educational intervention was structured into four modules to be administered in an equal number of face-to-face sessions, once per week and lasting three hours per session. The contents addressed per session were: (i) Definition of the disease, its signs and symptoms, (ii) Forms of the infection, (iii) Breeding sites of the vector, and (iv) Preventive actions to reduce the risk of infection. Each module was designed employing educational strategies, such as master classes, guided discussions, workshops, drawings, and use of a printed booklet ( https://drive.google.com/file/d/13MqOIgE6XrpMdaA4a4irFAnjseTdRsoJ/view?usp=drive_link), designed bearing in mind the observations made by indigenous authorities from the Emberá Katío community, who were consulted to know their perceptions and suggestions about the intervention. Each educational session began with a welcome greeting and assessment of prior knowledge, then the planned theme was developed and, finally, an activity was assigned to be developed at home and socialized during the following session. 

*Stage 2 - Content validation of the educational intervention through expert judgment*

Thirteen experts participated in this process. They met the inclusion criteria of being professional in any health science or social science discipline, having a graduate degree, and fulfilling one of the following two criteria: (i) having at least two years of experience in malaria prevention and treatment in teaching and research areas, or (ii) having work experience with indigenous communities. It was considered that the intervention had adequate content validity with the following Content Validity Indices per criteria: 0.83 in clarity, 0.90 in pertinence, 0.81 in relevance, and 0.88 in coherence. The observations and recommendations by the experts were considered for the intervention adjustments. The outcomes of this stage were described in detail and are published in a Nursing journal in Colombia.^14^

*Stage 3 - Quasi-experimental study with control group*

This type of study was chosen due to the randomization difficulty to assign the participants to the intervention and control groups due to the cultural characteristics and geographic location of the Emberá community; a situation that facilitated contamination with information from the study itself provided to the intervention group to those who would be assigned to the control group. Likewise, it was not possible to guarantee participant blinding because the informed consent detailed the activities that would be carried out in the intervention and control groups. It was also not possible to blind the evaluator, who was the principal researcher and knew the participants' membership in the groups. 

The study population was comprised by people who lived in the Alto Sinú region of the municipality of Tierralta, department of Córdoba (Colombia). The following defined the inclusion criteria: age ≥ 18 years, being an inhabitant of the region, and belonging to the Emberá Katío ethnic group. Exclusion criteria were: not attending one or more of the sessions programmed to receive the intervention, incomplete answer in at least 20% of the questionnaire, and voluntary withdrawal from the study. Initially, and through the social contact strategy, the leaders and senior indigenous authorities were summoned, through the Association of Councils of Alto Sinú. They granted permission to enter the area and suggested choosing the settlements of El Rosario and San Clemente from the eight that make up the indigenous reservation of Alto Sinú (Tierralta, Department of Córdoba, Colombia) due to having less traffic of actors in the armed conflict living in the area and, therefore, less risk to the safety of researchers and participants. This reason defined the sampling as intentional type. To calculate the sample difference between two proportions, the formula by Fleiss *et al*., was used,^15^ bearing in mind the statistical parameters: rate of individuals from the intervention group to the control group of 1:1, 95% significance level, and 80% power. The proportions of knowledge about malaria were assumed at 84% in the control group versus 96% in the group receiving an educational intervention for malaria prevention in the study by Alvarado *et al.*;^9^ under these conditions, the minimum sample size was 52 individuals per group. 

As for the assignment of the two indigenous communities to intervention or control groups, it was done by the coin toss method, leaving the San Clemente resettlement as the intervention group (IG) and El Rosario as the control group (CG). During the participants’ enrollment procedure, the indigenous leaders acted as mediators between the researcher and the members of the communities selected. During the time the research took place, both study groups continued receiving routine care, considering such as the set of activities provided regularly and in planned manner that health services providing institutions offer to their users.^16^ For this case, it was the care provided by the Municipal Health Secretariat from the municipality of Tierralta, consisting of health education, thick blood sample collection campaigns, and placement of posters in areas of greatest affluence. Additionally, the intervention group was administered the educational intervention in four sessions, each lasting three hours; the control group received one session with the summary of the content from the intervention upon completing the study. The activities conducted in each of the groups are detailed in the box below. 

**Table:** Description of the activities provided to the intervention and control groups

Group	Group	
Intervention	Control	Description
Enrollment	Enrollment	Prior to starting the intervention, informed consent was explained and signed, and sociodemographic data were collected.
FIRST KAP measurement	FIRST KAP measurement	All the participants had the first KAP measurement regarding malaria prevention.
Application of Module 1	Routine care	Week 1. Development of the first module of the educational intervention: *Knowing malaria*
Application of Module 2	Routine care	Week 2. Development of the second module of the educational intervention: *How can we get sick from malaria?*
Application of Module 3	Routine care	Week 3. Development of the third module of the educational intervention: *Where can the malaria mosquito grow?*
Application of Module 4	Routine care	Week 4. Development of the fourth module of the educational intervention: *How can I prevent malaria?*
Second KAP evaluation	Second KAP evaluation	Week 5. All the participants had the second KAP measurement regarding malaria prevention.
No activity	Application of the intervention summary	Week 6. The control group received the summary of the contents of the educational intervention during a single session and were given the booklet.

The instrument used to measure KAP was *The KAP Survey on addressing malaria in indigenous communities,* which is composed by 45 questions contained in seven components: 1-general sociodemographic data, 2-knowledge, 3-attitudes, 4-practices, 5-perceptions against malaria, 6-access to the health services network, and 7-community participation. This scale has been validated in indigenous population in Colombia, having a Cronbach’s alpha of 0.72.^17^ The instrument was applied with all the participants in both communities selected, through reading the questions individually. The intervention of an interpreter of the Emberá language was not necessary, because all the participants spoke and understood Spanish.

The information obtained in the study was analyzed with the SPSS program v.29. To compare between groups of the baseline sociodemographic variables and the KAPs evaluations at the PRE-PRE and POST-POST moments, Student’s t test was used for two independent samples for quantitative variables, and the Chi-squared test for qualitative variables. In the case of the latter, the Yates correction was applied if any of the expected values ​​were ≤ 5. To compare the PRE-POST intragroup difference of the KAPs, Wilcoxon's non-parametric signed-rank test for two related samples was used.

General Linear Models (GLM) of repeated measures were constructed to assess the effect of the educational intervention on the possible intervening variables to improve KAPs. Variables with intragroup PRE and POST intervention measurement were defined as repeated measures factor. The intra-subject factor was the group variable (0 = control and 1 = experimental); and, to control the effect of possible confusion, covariables: age, sex, years of education, and years living in the location were introduced into the models.

Given that the instrument ‘KAP survey on addressing malaria in indigenous communities*’* does not have a scoring system,^6^ it was decided add up the questions correctly answered in three of the components: knowledge (*n* = 8), attitudes (*n* = 6), and practices (*n* = 10). The same was not done with the items of the components access to the health services network and community participation because these are questions that are perceptions about activities conducted by individuals different from those surveyed; these questions were kept to preserve comparability with other studies where this instrument has been used.

The statistic to test the null hypothesis related with the effect of the time factor (PRE and POST intervention measurements) on improving KAPs in the study groups was Pillai’s trace, assuming a probability value < 0.05 to reject the null hypothesis and conclude that the explicative variable (intervention) has significant effect on the values of the response variables, PRE and POST intervention measures. Mauchly’s test of sphericity showed in all the GLM carried out in this research a value of 1.0, implying that the assumption of sphericity was fulfilled.^18^

The partial Eta squared was used to evaluate the size of the effect. This value varies from 0 to 1, where values closest to 1 indicate a higher proportion of variance that can be explained by a given variable in the model after taking into account the variance explained by the other variables; its interpretation is: small **(**0.01-0.05), medium (0.06-0.13), and large (≥ 0.14).^19^ Statistical significance was considered for all tests used in the statistical analysis if the probability value was < 0.05. A pilot test was conducted with 10% of the sample size (20 indigenous individuals who did not participate in the study groups) to evaluate preliminarily the intervention regarding possible difficulties to deploy the intervention and the key methodological aspects, like enrollment and information collection. The measurement instrument to evaluate necessary adjustments for its semantic adequacy was applied to the participants of this phase, identifying the need to include in the questions that had the word “malaria” the option “or paludism” because some people manifested knowing this disease by this name. 

After deploying the educational intervention in the experimental group, its acceptability was valued with 11 items that assess compliance of the categories: suitability (*n* = 2), convenience (*n* = 1), effectiveness (*n* = 2), health risk (*n* = 1), adherence (*n* = 1), and form of delivery (*n* = 4), proposed by Sidani and Braden (20). These questions were answered through self-report and in person by 20 of the 58 indigenous participants (34.5%). This survey used Likert-type response options, thus: 1-Not acceptable at all; 2-Somewhat acceptable; 3-Acceptable; 4-Quite acceptable; 5-Totally acceptable. It was found that each of the items evaluated a maximum average value of 5 and a minimum value of 4.

The Project was approved by the Research Ethics Committee of the Faculty of Nursing at Universidad de Antioquia through Act N.º CEI-FE 2021-37. During its execution, the ethical principles governing human research were guaranteed and, according with Resolution 8430 of 1993, it was classified as minimum-risk research. Informed signed consent was obtained from the participants and permission was provided by the Association of Indigenous Councils of Alto Sinú. Additionally, suggestions made by community members were taken into account during the planning of the educational intervention. This study was registered in ClinicalTrials.gov with Identifier: NCT06590597.

## Results

**Figure 1:**
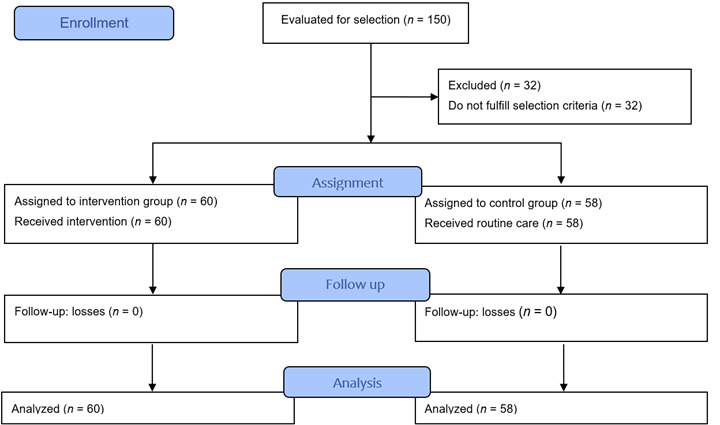
Flowchart of selection and retention of participants in the quasi-experimental study

During the enrollment carried out in the resettlements of El Rosario and San Clemente, 150 people interested in participating in the research were evaluated. Of this total, 32 were excluded for not fulfilling the established inclusion criteria, thus, 118 people participated (60 in the intervention group - resettlement of San Clemente - and 58 in the control group - resettlement of El Rosario-). There were no losses during follow up (Figure 1).

As per the sociodemographic characteristics of the participants, no statistically significant differences were found in most of the variables between the study groups; the exceptions were in some of the variables of household information. Table 1 shows that the following characteristics prevailed for all: average age of 37.43± 9.49 years (minimum = 20; maximum = 57), female sex (78%), common-law marital status (61.0%), and affiliation to the subsidized health regime (77.1%). With respect to schooling, the average number of years of study was 11.5±2.9 (minimum = 3; maximum = 18), and in relation to time in the location, the study found an average of 28.6±13.9 years (minimum = 3; maximum = 55). Regarding occupation, being a housewife prevailed (49.2%). In relation to information about the household, cement floors predominated (92.4%), but there were significant differences in the type of roof made of fiber cement tiles (IG = 51.7% and CG = 97.7%), and on the type of cement block wall (IG = 43.3% and CG = 94.8%). 

Regarding public utility services, all the households from both groups had electric power and natural gas and lacked sewage service. Significant difference was evidenced in the availability of internet service (IG = 40% and CG = 19%), likewise in the potable water service (IG = 75% and CG = 94.8%); with the main source of water for consumption being the rural aqueduct in both groups. Upon analyzing the information on the lifetime prevalence of malaria, it was of 39.8% for all the participants, with statistically significant difference (*p* < 0.001) between the groups (IG = 63.3% and CG = 15.5%). With respect to annual prevalence of malaria, it was estimated in IG = 3.3% and CG = 3.4%. None of the participants from the groups reported death of relatives due to malaria during their lifetime. 

**Table 1:** Description of the total sociodemographic variables and according to study group

Variables	Group	Group		Test statistic	*p*-value
	Intervention (*n* = 60)	Control (*n* = 58)	Total (*n* = 118)		
Age in years; Mean ±SD	36.1±9.9	38.8±8.9	37.43±9.49	-1.600‡	0.056
Female sex; *n* (%)	45 (75%)	47 (81%)	92 (78%)	0.625*	0.429
Marital status; *n* (%)				2.017*	0.365
Single	14 (23.3%)	11 (19%)	25 (21.2%)		
Married	13 (21.7%)	8 (13.8%)	21 (17.8%)		
Common-law	33 (55%)	39 (67.2%)	72 (61%)		
Health affiliation; *n* (%)				5.482*	0.064
Contributive	17 (28.3%)	8 (13.8%)	25 (21.2%)		
Beneficiary	0 (0%)	2 (3.4%)	2 (1.7%)		
Subsidized	43 (71.7%)	48 (82.8%)	91 (77.1%)		
Years of schooling; Average ±SD	11.7±3.0	11.2±2.9	11.5±2.9	0.831‡	0.204
Years in the location; Average ±SD	26.9±14.1	30.4±13.7	28.6±13.9	-1.352‡	0.90
Currently working *n* (%)	59 (98.3%)	58 (100%)	117 (99.2%)	0.000†	1.000
Occupation *n* (%)				8.424*	0.297
Agriculture	3 (5%)	9 (15.5%)	1 (0.8%)		
Cattle raising	3 (5%)	4 (6.9%)	7 (5.9%)		
Day laborer	2 (3.3%)	0 (0%)	2 (1.7%)		
Various jobs	5 (8.3%)	8 (13.8%)	13 (11.0%)		
Worker	2 (3.3%)	3 (5.2%)	5 (4.2%)		
Housewife	33 (55%)	26 (44.8%)	58 (49.2%)		
Other	12 (20%)	8 (13.8%)	20 (16.9%)		
Information about housing					
Type of roof; *n* (%)				27.797*	< 0.001
Plant or palm	10 (16.7%)	1 (1.7%)	11 (9.3%)		
Zinc	19 (31.7%)	2 (3.4%)	21 (17.8%)		
Fibrous cement	31 (51.7%)	55 (94.8%)	86 (72.9%)		
Type of wall; *n* (%)				39.360*	< 0.001
Wood	6 (10%)	3 (5.2%)	9 (7.6%)		
Cement block	26 (43.3%)	55 (94.8%)	81 (68.6%)		
Brick	27 (45%)	0 (0%)	27 (22.9%)		
Guadua	1 (1.7%)	0 (0%)	1 (0.8%)		
Type of floor; *n* (%)				0.411†	0.522
Cement	54 (90%)	55 (94.8%)	109 (92.4%)		
Wood	6 (10%)	3 (5.2%)	9 (7.6%)	-	-
Public services in the dwelling					
Natural gas	58 (96.7%)	56 (96.6%)	114 (96.6%)	< 0.001†	1
Electricity	60 (100%)	58 (100%)	118 (100%)	-	-
Internet at home	24 (40%)	11(19%)	35 (29.7%)	6.254*	0.012
Trash collection	0	0	-	-	-
Drinking water	45 (75%)	55 (94.8%)	100 (84.7%)	8.969*	0.003
Sewage	0	0	-	-	-
Origin of the water; *n* (%)				8.969*	0.003
Rural aqueduct	45 (75%)	55 (94.8%)	100 (84.7%)		
Well	15 (25%)	3 (5.2%)	18 (15.3%)		

*: Pearson’s (2; †:((2 with Yates continuity correction; ‡: Student’s t

### POST-test results between groups

Upon applying the second measurement, there was statistically significant difference in most of the KAP variables, with the percentages being better in the IG compared with the CG, for which emphasis will be made on aspects where there was no difference.

Knowledge. After applying the second measurement, statistically significant difference was observed in most of the variables in this domain, with the percentages in the IG being better, except for the variables: *recognition of bodily pain as a symptom of malaria* (IG = 95% vs. CG = 86.2%; *p* = 0.101*), malaria is cured: with the treatment provided by the health institution* (IG = 100% vs. CG = 96.6%.; *p* = 0.461) or by *going to a pharmacy* (IG = 0% vs. CG = 1.7%; *p* = 0.986); and in the questions: *what can be done for a person not to get malaria,* in the items of: using insect netting (IG = 100% vs. CG = 98.3%; *p* = 0.986), fumigate with insecticides (IG = 100% vs. CG = 98.3%; *p* = 0.986); and in the item that the mayor should also help to prevent malaria (IG = 5% vs. CG = 1.7%; *p* = 0.635). 

Attitudes. It was observed that in two of the six questions in this domain no statistically significant difference was noted, although clarifying that both came with high percentages since the PRE-test measurement: *do you agree with spraying insecticides in your house?* (IG = 100% vs. CG = 98.3%; *p* = 0.986) and *do you consider that malaria is a problem for you and your family?* (IG = 100%; vs. CG = 98.3%; *p* = 0.986).

Practices. Only this question of the ten that make up this domain did not have statistical difference: *do you use artificial repellents against mosquitos?* (IG = 11.7% vs. CG = 1.7%; *p* = 0.075). 

### Intragroup PRE- and POST-test results

Knowledge. All participants from both groups knew what malaria was, both during the PRE-test and POST-test. In the rest of the questions from this section, statistically significant difference was found exclusively in the intervention group, indicating improved knowledge in the variables: *mechanism of malaria transmission* - through water, person to person, and through mosquito bite - (PRE = 46.7% vs. POST = 100%; *p* < 0.001), *symptoms a person with malaria may have* - pain, on the body, weakness and fatigue, chills and vomiting - (PRE = 35% vs. POST = 100%; *p* < 0.001), *tests made to know if someone has malaria* - blood sample - (PRE = 48.3% vs. POST = 100%; *p* < 0.001), *how is malaria cured* - taking the treatment provided by the health institution- (PRE = 65% vs. POST = 100%; *p* < 0.001), *actions to prevent people from getting malaria* - avoid stagnant waters, use insect netting over beds, keep the household clean, avoid going out at night, use clothing for protection - (PRE = 45.0% vs. POST = 93.3%; *p <* 0.001), *who should prevent malaria* - the family, neighbors, community in general - (PRE = 53.4% vs. POST = 95%; *p* < 0.001) and, finally, in the variable: *do you know the name of the mosquito that transmits malaria?* (PRE = 13.3% vs. POST = 91.7%; *p* < 0.001).

Attitudes. There was exclusive improvement in the intervention group for the variables: *do you think a person with malaria should take the whole treatment?* (PRE = 70.0% vs. POST = 100%; *p* < 0.001); *do you agree with spraying insecticides in the house* (PRE = 61.7% vs. POST = 100%; *p <* 0.001); *do you agree with the following activities to prevent malaria?* (statistical difference in all activities) (PRE = 36.7% vs. POST = 100%; *p* < 0.001); and, finally in the variable: *do you consider that malaria is a problem for you and your family?* (PRE = 68.3% vs. POST = 100%; *p* < 0.001) In addition, the attitude was maintained related to the variable: *when someone has malaria where should they go?* Of respondents, 75% preferred health services 75% and 15% consults with the Jaibaná.

Practices. It was possible to note exclusive improvement in the IG for the items that make up this domain, except for the use of natural repellents and burning of plants to repel mosquitos, each of them with 11.7% in both measurements. 

### Results of GLM

In all the GLM of repeated measures conducted for the score factors of knowledge, attitudes, practices, and for total KAP, the assumption of sphericity was fulfilled. All the models were significant (*p* < 0.01). The size of the effect, measured by the partial Eta squared statistic, indicating the change in the sum of the questions correctly answered in the educational intervention, was 91% in knowledge, 48.9% in attitudes, 85.8% in practices, and 93.5% of the total KAPs (Table 2).

**Table 2:** Tests of intra-subject effects in the Linear Regression Models of repeated measures for factor scores of knowledge, attitudes, practices, and total KAP

Origin	Type III sum of squares		GL	Root mean square	F	***p*-value**	Partial Eta Squared
Factor = Knowledge score							
Factor	Assumed sphericity	2.36	1	2.362	7.22	0.008	0.061
Factor * Group	Assumed sphericity	370.182	1	370.182	1132.360	< 0.001	0.910
Error (factor)	Assumed sphericity	36.614	112	0.327	-	-	-
Factor = Attitude score							
Factor	Assumed sphericity	0.063	1	0.063	0.313	0.577	0.003
Factor * Group	Assumed sphericity	21.436	1	21.436	107.340	< 0.001	0.489
Error (factor)	Assumed sphericity	22.367	112	0.200	-	-	0.200
Factor = Practices score							
Factor	Assumed sphericity	0.205	1	0.205	0.678	0.412	0.006
Factor * Group	Assumed sphericity	204.168	1	204.168	676.297	< 0.001	0.858
Error (factor)	Assumed sphericity	33.812	112	0.302	-	-	-
Factor = Total KAP score							
Factor	Assumed sphericity	3.025	1	3.025	3.327	0.071	0.029
Factor * Group	Assumed sphericity	1456.090	1	1456.090	1601.598	< 0.001	0.935
Error (factor)	Assumed sphericity	101.825	112	0.909	-	-	-

Regarding pairwise comparisons, it was found that statistically significant differences exist among the average total of questions correctly answered, as well as for each of the factors, between the IG and CG, with average scores being higher in the IG (Table 3).

**Table 3:** Pairwise comparisons of the difference in the number of questions correctly answered per factor between the intervention group and the control group

Factor	Number of questions	Differences of intra-group means	Differences of intra-group means	Difference of means	***p*-value** ^*^	95% CI difference^*^	95% CI difference^*^
		IG	CG			LL	UL
Knowledge score	8	5.66±0.07	3.12±0.08	2.54±0.07^*^	< 0.001	2.39	2.68
Attitude score	6	3.57±0.04	3.00±0.07	0.54±0.07*	< 0.001	0.37	0.66
Practices score	10	5.32±0.52	3.41±0.06	1.91±0.07*	< 0.001	1.76	2.10
Total KAP score	24	14.50±0.15	9.58±0.15	4.92±0.12*	< 0.001	4.66	5.17

(*) Bonferroni adjustment; (95% CI) 95% Confidence Interval; (LL) Lower limit; (UL) Upper limit.

## Discussion

This research found that in most baseline sociodemographic conditions no statistically significant differences existed between the study groups, except for some variables related with housing, which permitted their comparison. Majority participation by women in the study and their occupation as housewives can be explained by the distribution of activities within the families. In this dynamic, men are dedicated to obtaining economic resources and food, as well as participating in political activities, while women are in charge of caring for the children, animals, and the household.^21^ It is possible that these responsibilities make them more prone to participating in these types of events and feeling responsible for putting into practice at home what has been learned. With respect to education, the average global number of years of schooling was 11.5 years, which in Colombia would be related completing secondary education,^22^ being higher than that reported in indigenous population in Colombia, where almost half the people had as maximum level complete primary education.^5^

Regarding the housing characteristics, cement floors predominated with differences in the types of roofs and walls between the groups. In the control group, greater use of fiber cement tiles and cement blocks was found. These disparities could be explained by the proximity of the El Rosario resettlement (control group) to the urban perimeter, which facilitates obtaining these types of materials. This also evidences the westernization of constructions in these communities, which could be related with the relocation of the Emberá Katío people due to the construction of the Urra I hydroelectric. This relocation meant uprooting their lands, customs, and ancestral knowledge.^23^ This finding also agrees with that described by the National Administrative Department of Statistics (DANE, for the term in Spanish),^5^ which shows that 60.8% of the dwellings with indigenous heads of household were built as houses, while only 30% lived in traditional indigenous housing. With regards to public utility services, greater availability of Internet service was found in the intervention group and drinking water in the control group. The foregoing is related with that described by some inhabitants of the zone, who indicated that currently projects are being executed, such as satellite Internet, rural aqueducts, and improvements in tertiary roads, which are more advanced in some populations than in others. 

With respect to information on cases of malaria, the experimental group reported a lifetime prevalence four times that reported by the control group. Nonetheless, the annual prevalence was < 4% in both groups. This difference may be justified by the lower permanent availability of drinking water in the intervention group, which obligates them to store water in containers that are possible breeding sites of the malaria vector.

Moving on to the information from the repeated measures GLMs, the positive effect of the educational intervention was shown, which explained the variance in improving the score of correct answers by 91% in knowledge, 49% in attitudes, 85% in practices, and 93% in the total KAP score. These results coincide with those reported by other studies that observed significant changes in KAP scores with respect to interventions for malaria prevention in indigenous communities.^7^^,^^9^^,^^24^

Although the results in this study evidence the benefits of the intervention to improve KAPs in malaria prevention, it is important to note that lower changes were noted in the scores in the attitudes factor, a situation also found by Balami *et al*.^25^ When specifically reviewing this factor in our study, it was found that questions about whether they agreed with the actions of filling puddles with soil and drilling holes in objects that could retain water, among the measures to prevent malaria, these improved little. Likewise, the practices factor also showed little change in using fans to repel mosquitos, applying artificial repellents, and wearing clothing to protect against bites. This finding could not be contrasted with other research, given that results reported by authors who conducted educational interventions to improve KAPs in malaria prevention in indigenous communities did not document specifically the actions evaluated in the factors of attitudes and practices, pre and post-intervention.^7^^,^^9^^,^^24^

However, researchers have indicated that human behavior may be influenced by social, cultural, economic, and political factors, which - in turn - can increase or diminish the risk of suffering a disease.^6^ In this sense, the low percentages observed in activities related with the attitudes factor could be explained by the influence of these factors. At the same time, the little change in activities, like using fans, applying artificial repellents, and wearing clothing that protects against bites could be attributed to the low wages earned by families in the area.^5^ This condition reduces the possibility to acquire elements, like fans, commercial repellents, and clothing with special characteristics to avoid the vector’s bite. The foregoing creates a scenario in which the different players in charge of actions to promote and maintain health must adopt strategies aimed at strengthening these types of activities through supporting the acquisition and teaching the use of implements mentioned in malaria prevention. Further, the finding on the use of apparel coincides with the practice of wearing traditional Emberá clothing, a custom kept by some members of the community.^26^

Also, with respect to the practices factor, in the experimental group and the control group, it was possible to identify that some participants manifested the practice of burning leaves from plants, like ‘matarratón’ (*Gliricidia sepium*) to repel mosquitos and as natural repellents. This practice was maintained in both groups both during the pre and post-test. This is related to the customs and cultural roots of the Emberá that are still present in the area, especially with the figure of the Jaibaná and use of traditional medicine, which includes using plants to cure and prevent diseases transmitted by vectors.^26^^,^^27^

These results coincide with that stated by other authors^28^^,^^29^ regarding the need to understand health phenomena and care practices within the cultural context of people, as well as that proposed by health organizations, like the PAHO^12^, an entity that recognizes the importance of facing ethnic, social, and cultural diversities, and of bridging existing gaps when considering the peculiarities and needs of these groups by the health systems of its member states. Due to the aforementioned, the work of nursing professionals should be aimed at guaranteeing equitable access to health services, respecting values and cultural beliefs, safeguarding the needs of these population groups.^30^^,^^31^

Under this vision, levels of self-efficacy and cultural competence of nursing professionals must be improved in the Colombian context, through promoting cultural knowledge as a mechanism to promote culturally competent care.^32^ The challenge continues being the need to create protocols and implementation plans on knowledge, sensitivity, awareness and cultural competence, as well as these types of educational interventions. This is why the importance is highlighted of the role of the nursing discipline in the face of the phenomenon of globalization of health care, where it is increasingly expected for professionals in the area globally to come in contact with patients, families, and colleagues from diverse cultures and origins,^33^ interaction that represents humanized care and adjusted to the cultural peculiarities of each person and community.

Finally, this research has implications for the nursing practice, given that the knowledge generated herein can serve as referent when planning care in similar contexts as that developed by this study. Besides, this research contributes a validated and effective educational intervention to improve the KAP regarding malaria prevention in a vulnerable group, like the Emberá Katío community from the municipality of Tierralta in Córdoba, Colombia. Moreover, inclusion of theoretical assumptions from transcultural nursing represents a substantial contribution to the nursing discipline in research methodological aspects, as well as to the development of care actions culturally sensitive with the reality of people.

To conclude, it can be stated that the educational intervention was effective in improving knowledge, attitudes, and practices for malaria prevention in an indigenous population of the Emberá Katío ethnicity from the department of Córdoba, Colombia. Nevertheless, it is needed to evaluate its effectiveness in similar contexts and continue the design of research that address specific aspects of the domains of attitudes and practices, such as the elimination of vector breeding sites and the use of repellents. 
